# Active spheres induce Marangoni flows that drive collective dynamics

**DOI:** 10.1140/epje/s10189-020-00006-5

**Published:** 2021-03-08

**Authors:** Martin Wittmann, Mihail N. Popescu, Alvaro Domínguez, Juliane Simmchen

**Affiliations:** 1 Technical University Dresden, Zellescher Weg 19, 01069 Dresden, Germany; 2 Max Planck Institute for Intelligent Systems, Heisenbergstr. 3, 70569 Stuttgart, Germany; 3 Física Teórica, Universidad de Sevilla, Apdo. 1065, 41080 Sevilla, Spain; 4 Instituto Carlos I de Física Teórica y Computacional, 18071 Granada, Spain

## Abstract

**Abstract:**

For monolayers of chemically active particles at a fluid interface, collective dynamics is predicted to arise owing to activity-induced Marangoni flow even if the particles are not self-propelled. Here, we test this prediction by employing a monolayer of spherically symmetric active $\hbox {TiO}_2$ particles located at an oil–water interface with or without addition of a nonionic surfactant. Due to the spherical symmetry, an individual particle does not self-propel. However, the gradients produced by the photochemical fuel degradation give rise to long-ranged Marangoni flows. For the case in which surfactant is added to the system, we indeed observe the emergence of collective motion, with dynamics dependent on the particle coverage of the monolayer. The experimental observations are discussed within the framework of a simple theoretical mean-field model.

**Graphic abstract:**

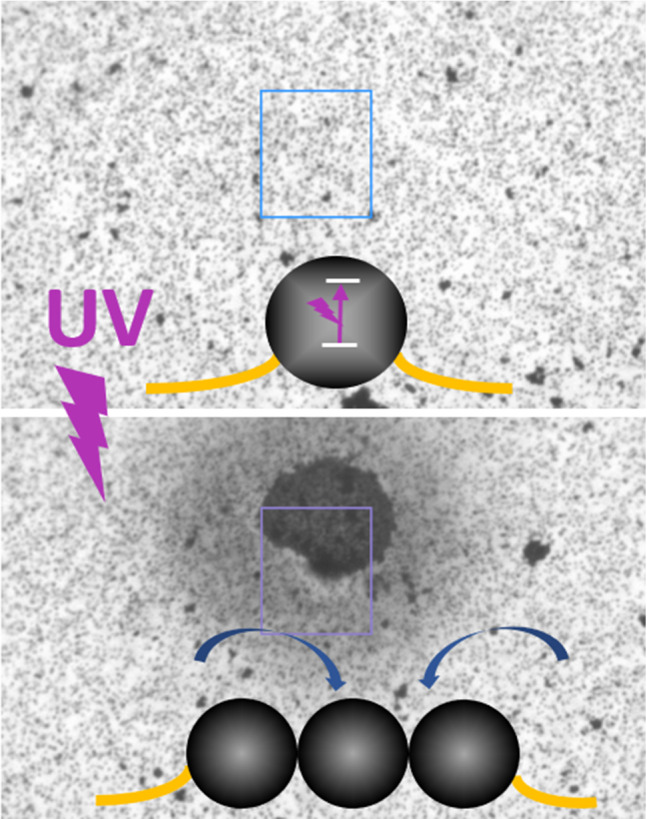

## Introduction

The challenge of endowing micro- and nano-sized particles with motility, without applying external forces or torques, has received significant interest from both perspectives of applied and basic science (see, e.g., the insightful reviews in Refs. [1–3]). As noted in the classic paper of Purcell [4], the issue of motility of such micrometer-sized objects in Newtonian liquids of viscosity similar to that of water is particularly intriguing and interesting. For such systems, the Reynolds number is very low and thus the hydrodynamics is governed by Stokes equations, while the motion of the object is in the overdamped regime. Accordingly, such objects cannot rely on inertia to move steadily (see also the review in Ref. [5]). Hence, achieving steady directional motion of colloidal particles requires strategies to break the time-reversal symmetry of the Stokes equations [4].

**Fig. 1:**
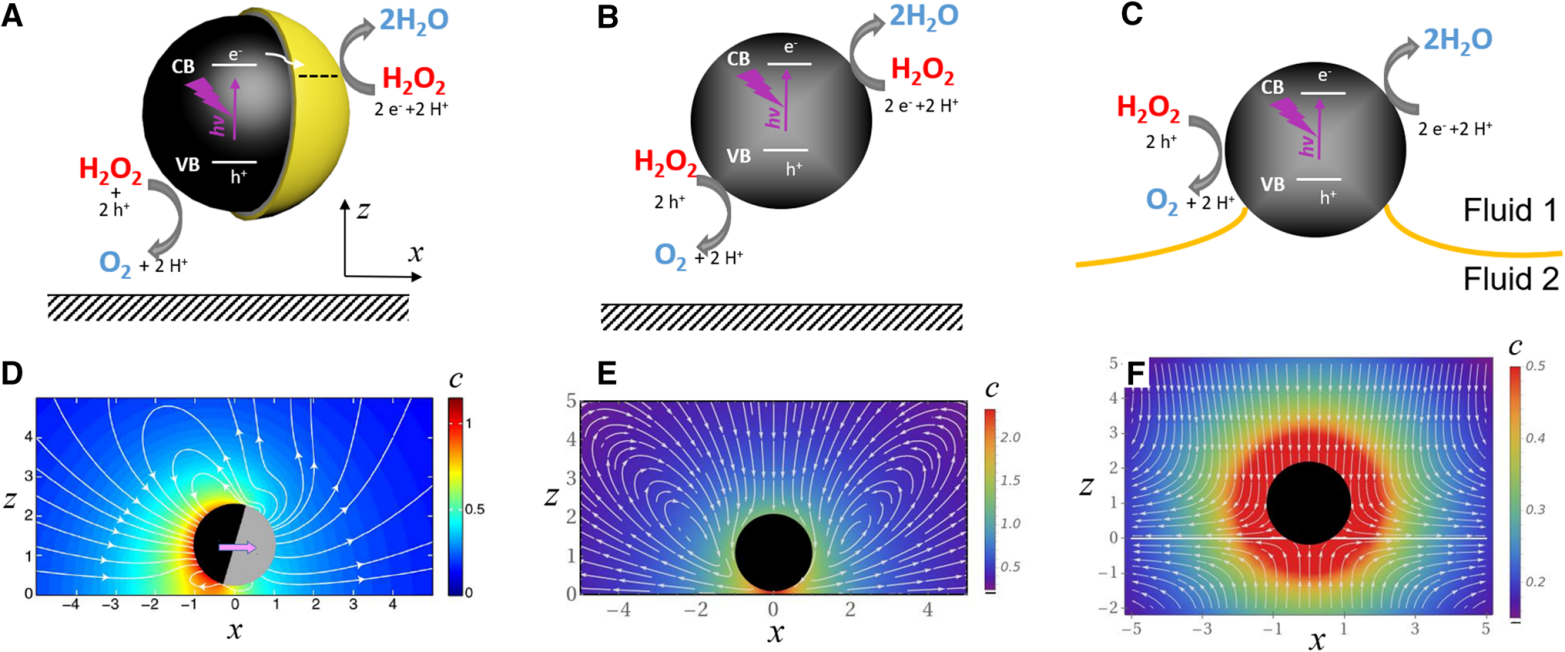
**a**–**c** Schematic representations of a Janus (**a**) and isotropic (**b**, **c**) active particles, respectively, in the vicinity of a wall (**a**, **b**) or at a fluid interface (**c**). The chemical activity is illustrated via the example of a semiconductor photocatalyst promoting, upon suitable illumination, the decomposition of hydrogen peroxide ($\hbox {H}_2\hbox {O}_2$) into water and oxygen. **d**–**f** Examples of the calculated concentration *c* of reaction product (color coded, in arbitrary units) and hydrodynamic flow (streamlines, white) (color figure online)

One direction which has been intensely pursued since the first reports of such particles [6, 7] is making Janus structures. These have one part of the surface active (chemically or thermally), while the other is inert (see the left panels in Fig. 1). Variations in activity along the surface generate spatial inhomogeneities in the chemical composition (or the temperature) of the surrounding suspension. Motility then emerges via, e.g., self-phoresis driven by the gradients of these self-generated inhomogeneities [8–15]. The motion of Janus particles, either in unbounded fluid or in the vicinity of solid–liquid interfaces, has been subject of numerous theoretical and experimental studies. Detailed and insightful reviews of such studies can be found, e.g., in Refs. [2, 16, 17]. The question of the self-phoresis of active Janus particles near, or trapped at, interfaces between two fluid phases, from now on referred to as *fluid interfaces*, has been recently tackled both experimentally [18–23] and theoretically [20, 24–26].

An interesting aspect specific to a fluid interface is that the interface itself can respond to the activity of the particles due to the locally induced changes in surface tension, which give rise to Marangoni stresses. For a Janus particle trapped at the fluid interface, these can directly drive the motion of the particle along the interface [27–30]. Furthermore, the Marangoni flows can couple back to the motion of a self-phoretic active Janus particle located *near* the interface [31]. Concerning collective effects, for “carpets” of self-propelled particles at a fluid interface the interplay between motility and induced Marangoni flows has been shown to possibly induce instabilities and the breakup of thin films [32].

As can be inferred from the discussion above, most of the studies of active particles have focused on particles for which self-motility is an intrinsic property. The case of chemically or thermally active particles which lack motility when isolated, but can set in motion when placed near confining surfaces or fluid interfaces, has been somewhat less explored. It was predicted that near a fluid interface either self-phoresis [26] or a self-induced Marangoni flow [33, 34] (as shown schematically in the rightmost pannel of Fig. 1) can move the particle toward or away from the planar interface.

When one of these particles with uniform activity over its surface is trapped at or near a planar interface, there cannot exist any “in-plane” motion, because of the radial symmetry of the in-plane Marangoni flow induced by the particle (see the rightmost pannel of Fig. 1) (unless there is a mechanism for an in-plane symmetry breaking along the interface, such as, e.g., the spontaneous autophoresis at large Péclet numbers discussed by Ref. [35]). However, when a collection of such particles forms a monolayer, the radial symmetry of the in-plane hydrodynamic flow is broken by the presence of the other particles^1^. The superposition of the in-plane components of the Marangoni flows induced by each particle becomes an effective pair interaction within the monolayer [34, 37]; this is actually a hydrodynamic interaction inasmuch as it is due to fluid flow, albeit sourced by the interfacial response, rather than by the motion of the particles. Consequently, inhomogeneities in the distribution of active particles within the monolayer induce motion of the— otherwise immotile—particles. Therefore, activity-induced collective dynamics within the monolayer may occur in spite of the absence of single-particle self-motility. As discussed in [37, 38], at least for simple models it can be shown that the state with uniform distribution of particles is unstable against perturbations, and collective dynamics sets in driven by the response of the interface in form of Marangoni stresses.^2^

**Fig. 2:**
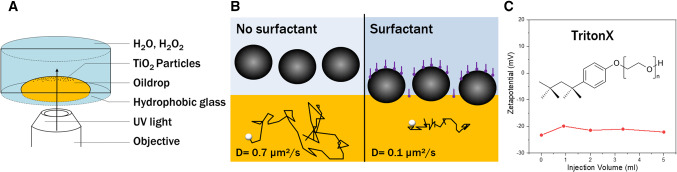
**a** Schematic representation of the experimental setup. **b** Schematic representation of the expected location of the particles (based on the measured in-plane diffusion coefficient) relative to the oil (yellow)–water (blue) fluid interface, without or with surfactant added, respectively. The broken lines show typical tracked in-plane trajectories of an inactive particle; the corresponding values of the in-plane diffusion coefficient *D* are shown. **c** The zeta potential of particles as a function of added surfactant (TritonX). The adsorption of the neutral surfactant molecules at the particle’s surface does not alter the charge conditions, which is in contrast to the case of using anionic or cationic surfactants (see Fig. 9 in “Appendix B”) (color figure online)

In general, the dynamics within such active monolayers involves the competition between the Marangoni effective interaction and other interaction, e.g., the direct forces that the particles exert on each other. For example, the issue of stability of a monolayer under the competition between the self-induced Marangoni flows and the weight-induced capillary attraction, both of which are long ranged (they decay with the distance *r* between the particles as $r^{-1}$, for *r* smaller than the capillary length), was addressed in Ref. [37]. The existence of stationary states sustained by the competition between the effective Marangoni interaction and a short-ranged repulsion was considered in Ref. [40]. It has been shown that in this case stationary states can emerge, with a radial “onion-like” structure (from a very dense, solid-like center to a dilute, gas-like rim) in the particle distribution induced by the interparticle repulsion within the monolayer, and the hydrodynamic flow within the fluid. The estimates for the length scale of such spatial structures [40], in agreement with the recently reported experimental setups involving monolayers of active colloids near (or at) fluid interfaces [19–21, 41], suggest that experimental validation of those theoretical predictions may be possible in the case of a water–air interface and volatile products of the chemical activity^3^. When the Marangoni flows are strong ($> \upmu$m/s), which can be the case at an oil–water interface, the mean-field analysis in Ref. [40] cannot be *a priori* justified, and interparticle correlations may play a significant role. This hints to the exciting possibility of a much richer phenomenology of collective dynamics emerging in such systems.

Motivated by these observations, we designed and carried out a study of the emergence of collective dynamics in a monolayer of chemically active spherical particles sedimented at an oil–water interface in the presence of a nonionic surfactant. Subsequently, the results are discussed and interpreted in the context of previously proposed simple theoretical models of activity-induced Marangoni flows [33, 34, 37, 38, 40].

## Model system and experimental setup

As model active particles we use photocatalytically active colloids based on spherical, isotropic $\hbox {TiO}_2$ particles with an average size of 700 nm [42]. Their chemical activity consists in promoting the photocatalytic degradation of hydrogen peroxide, $\hbox {H}_2\hbox {O}_2$, upon UV illumination (see the schematic in Fig. 1c). The use of photocatalysis has the significant advantage that the chemical activity can be switched on and off despite the fuel ($\hbox {H}_2\hbox {O}_2$) being present in the solution. In previous studies [43] we have shown that very efficient self-motile Janus colloids (Fig. 1a) can be made by half-covering such $\hbox {TiO}_2$ particles with a thin layer of a metal (e.g., Cu), which alters the reaction rate locally. Without this additional metal layer, only enhanced Brownian motion is observed (see “Appendix B”). When the intensity of UV illumination is sufficiently high, a fraction of the particles lift off from their sedimentation location either over a solid wall or over a fluid oil–water interface.

In order to test the emergence of activity-induced Marangoni dynamics with this kind of active particles, we designed a specific experimental setup (Fig. 2a). It consists of a cylindrical cell filled with an aqueous solution of $\hbox {H}_2\hbox {O}_2$ (1 %). This reservoir offers a sufficient quantity of fuel in order to guarantee a stable peroxide concentration over the duration of an experiment (in the order of several minutes). The bottom wall was functionalized with hexadecyltrimethoxysilane in order to achieve hydrophobic properties and to enhance the tendency of silicon oil to wet it. We used silicon oil of viscosity 1000 cSt and mass density 970 $\mathrm {kg/m^3}$, which is slightly lower than those of water (1000 $\mathrm {kg/m^3}$) and hydrogen peroxide (1100 $\mathrm {kg/m^3}$). An oil–water interface is created by carefully depositing with a cannula a drop of oil on the hydrophobic bottom wall of the cell. Despite the density mismatch, the wetting forces prevent the drop from lifting off by buoyancy and, at the same time, ensure a pancake-like shape of the drop, which has proven difficult to achieve using microfluidic chambers [41]. These combined effects enable a fluid interface that is flat to a good approximation and allows imaging over an extended area, which is crucial for the optical microscopy observations. When $\hbox {TiO}_2$ particles are added into the cell, they sediment and a monolayer is formed near this quasi-flat fluid interface which will be the focus of our analysis. In absence of the drop of oil the monolayer forms above the substrate solid wall.

Once the cell has been prepared, it is placed under the microscope and the illumination with UV light is performed through the objective, from below. The illuminated area has an almost square shape, and its dimensions can be varied to a certain extent. For this study, we fixed it to a rectangular area of about 1500 ${\upmu \mathrm{m}^2}$. A consequence of this setup is that only the particles that lie inside this area become chemically active. The rest of the particles are expected to remain inactive and they serve as passive tracers of the flows arising in the aqueous phase.

## Results and discussion

### Observation of collective dynamics

First, in order to rule out that the UV radiation *per se* might cause changes of the fluid interface that would induce flows, we tested the setup with passive $\hbox {SiO}_2$ particles (see Fig. 6 in “Appendix B”). We observed indeed no macroscopic or collective motion, apart from the expected Brownian fluctuations. Second, we tested the setup with $\hbox {TiO}_2$ particles in the absence of either fuel ($\hbox {H}_2\hbox {O}_2$) or UV illumination. Only Brownian motion of the particles was observed. Accordingly, from these we infer that particle activity is a prerequisite for collective motion.

However, when the particles in the cell without surfactant become active upon exposition to both fuel and UV light, again no collective motion is detected (see Fig. 7 in “Appendix B”). We only observe, as already remarked in Sec. 2, that some particles within the irradiated area manage to escape the focus plane by drifting in the vertical direction away from the fluid interface. This observation indicates that the particles reside close to the fluid interface, without actually being trapped by it. Indeed, it is known that pure hydrophilic particles generally do not show much adsorption onto a fluid interface [44].

Our interpretation of the lack of observable collective motion is that the dynamics may be too weak to be detected, with velocities that fall under the experimental resolution (well below $0.1\,\upmu \mathrm {m/s}$). Therefore, in order to enhance the Marangoni flows, we added a surfactant to the aqueous phase with the goal of trapping the particles at the interface, so that a cumulative effect arises due to the superposition of the Marangoni flows by each particle. (The addition of surfactant serves to overcome the energy barrier that inhibits the attachment of particles to the fluid interface, thus allowing the control of the positioning of the particles relative to the interface [45].) The surfactant addition might also result in an increase in the responsiveness of the interface by changing the surface tension; however, there is no direct way of measuring this effect.

After testing different types of surfactants, we opted for TritonX, a nonionic surfactant that does not significantly influence the zeta potential of the particles in order to keep the charge conditions constant (see Fig. 9 in “Appendix B”). In the absence of surfactant and irradiation, the in-plane diffusion coefficient of the particles sedimented on the fluid interface is about $0.7\, \upmu \mathrm {m^2/s}$, which is comparable to the value when the particles reside on a solid substrate (i.e., in absence of the drop of oil). After addition of TritonX, the diffusion coefficient decreased significantly to $0.1\, \upmu \mathrm {m^2/s}$. This decrease is due to the influence of the higher viscosity in the silicon oil phase [46]. Corroborated by the absence of liftoff when the particles are active, this indicates that the particles are effectively trapped at the interface.

**Fig. 3:**
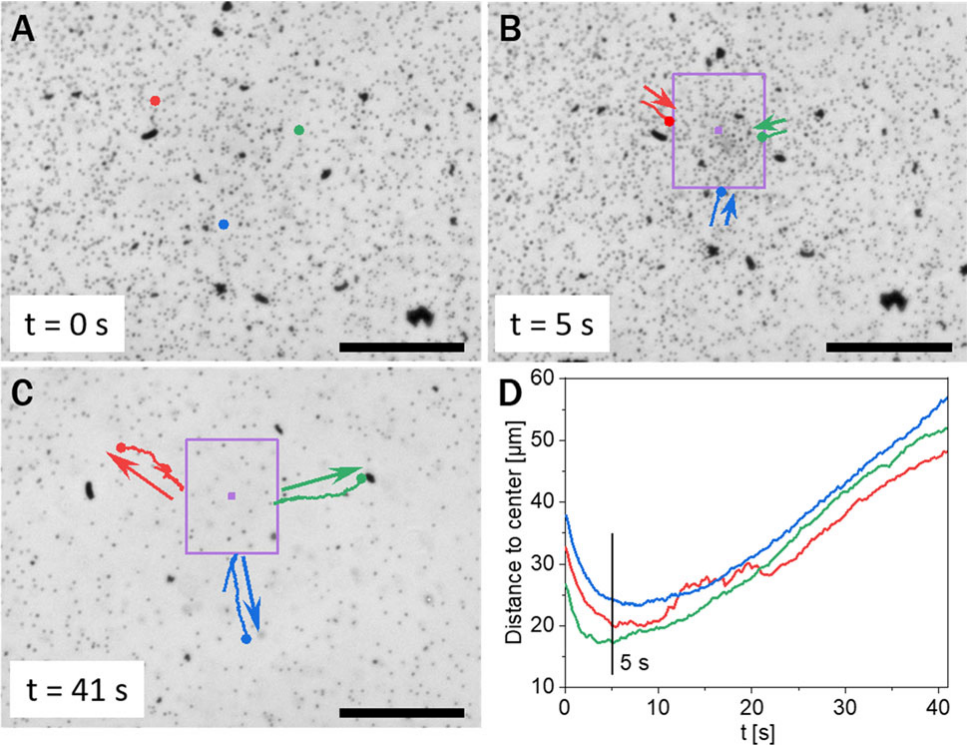
Behavior at low areal fraction: **a** Before onset of illumination the particles are uniformly distributed over the interface. **b** When UV irradiation is on (the violet square indicates the illuminated area, but the camera is not sensitive to UV light), an inward flow sets in, bringing particles toward (and into) the irradiated area. **c** The flow reverses direction after approximately 5 s; the outward flowing induces a depletion of particles from the illuminated area. **d** The distance to the center of the illuminated area for some tracked particles (the trajectories in **b** and **c**) is plotted as a function of the time after UV irradiation is switched on. (The scale bars correspond to 50 $\upmu$m.) (color figure online)

**Fig. 4:**
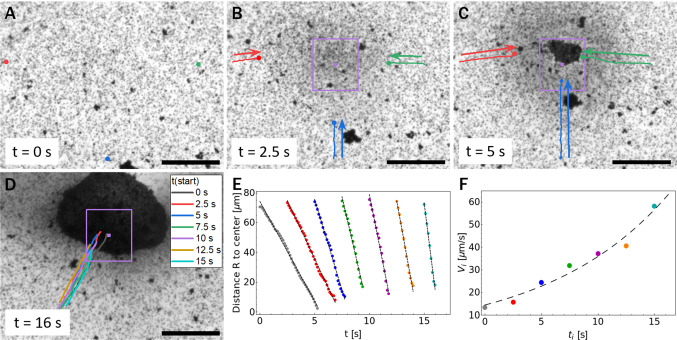
Behavior at high areal fraction: **a** Before onset of illumination the particles are uniformly distributed over the surface. **b** When UV irradiation is on (the violet square indicates the illuminated area), an inward flow sets in and brings particles toward (and into) the illuminated area. **c** Formation of a cluster within the illuminated region. **d** As long as the UV light remains on, the flow is maintained and the cluster grows. **e** The distance to the center of the illuminated area for the tracked particles (trajectories shown in panel **d** as a function of the time after the UV irradiation is switched on. The straight lines represent a fit to a motion with constant velocity $V_i$. **f** Plot of the velocity magnitude $|V_i|$ of a particle which at time $t_i$ (after illumination is turned on) is at a given distance $R_i$ to the center of the illuminated area (in this example, $R_i\approx 70\,\upmu \mathrm {m}$). The dashed line is the fit by Eq. (4). (The scale bars correspond to 50 $\upmu$m.) (color figure online)

**Fig. 6:**
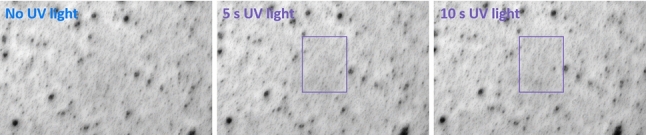
Passive $\hbox {SiO}_2$ particles in a solution of TritonX and $\hbox {H}_2\hbox {O}_2$ on oil–water interface. Left: before UV irradiation. Middle: after 5 s of UV irradiation. Right: after 10 s of UV irradiation

**Fig. 7:**
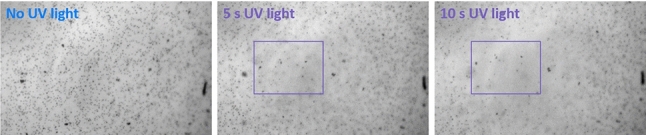
$\hbox {TiO}_2$ particles without addition of surfactant on oil–water interface. Left: before UV irradiation. Middle: after 5 s of UV irradiation. Right: after 10 s of UV irradiation

**Fig. 9:**
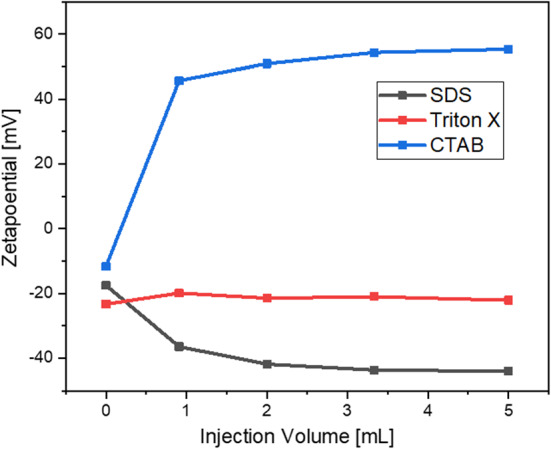
Zeta potential titrations of $\hbox {TiO}_2$ particles with different surfactants (SDS, CTAB and TritonX)

**Fig. 10:**
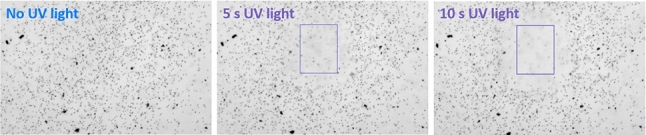
$\hbox {TiO}_2$ particles without surfactant on a solid substrate. Left: before UV irradiation. Middle: after 5 s of UV irradiation. Right: after 10 s of UV irradiation

**Fig. 11:**
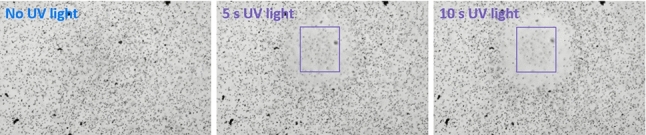
$\hbox {TiO}_2$ particles in a solution of TritonX on a solid substrate. Left: before UV irradiation. Middle: after 5 s of UV irradiation. Right: after 10 s of UV irradiation

Therefore, we repeated the experiment with active $\hbox {TiO}_2$ particles in the presence of a low (0.05 wt%) concentration of TritonX. Now we observe the emergence of collective flows of particles in the plane of the interface (Figs. 3 and 4). In order to support that this is due to a Marangoni flow, we tested that, when the particles reside on a solid substrate, the addition of surfactant does not induce any in-plane collective motion (see Figs. 10 and 11 in “Appendix B”), which convincingly rules out the chemical (“phoretic”) interactions as the source of the observed behavior. To dismiss the assumption of thermally driven Marangoni flows (induced by a hypothetical heating of the titania particles due to UV absorption), we compared the behavior of UV-illuminated active particles at the oil–(water plus peroxide) interface in the presence and absence of surfactant and found collective dynamics only in the first case. Moreover, these same observations allow us to also rule out mechanisms based on Marangoni stresses due to some trace amounts of contaminants at the interface, a scenario invoked by Ref. [30] in the context of the motion of heated Au@$\hbox {SiO}_2$ Janus particles at an oil–water interface.

In summary, we have identified the minimal ingredients required for the emergence of significant activity-induced collective dynamics with spherically symmetric particles in our experimental setup: besides the photocatalytic particles fueled by hydrogen peroxide and a fluid interface in our specific conditions, also a surfactant is necessary in order to generate observable Marangoni flows.

### Dependence of the collective dynamics on the areal density of particles

The photocatalytic activity of the $\hbox {TiO}_2$ particles scales with light intensity and fuel concentration, both leading to an increase in product concentration. We also confirmed that the behavior of the system depends on these two factors, resembling strongly to what is presented here. However, it turns out that the impact of the areal density of particles in the monolayer on the emerging dynamics is much more significant. For this reason, we decided to keep the light intensity and the fuel concentration fixed and, for the rest of this study, to investigate the dynamics at different particle densities.

At low particle areal density (12 % surface coverage), we observe the effect displayed in Fig. 3. Before irradiation with UV light, the particles are uniformly distributed over the fluid interface (Fig. 3a). Once the UV irradiation in the marked area is switched on, a collective flow toward the center of the illuminated region is observed (Fig. 3b). After a few seconds, and without any changes in the experimental parameters, the direction of the flow reverses (Fig. 3c). A steady state is not reached within the experimental times. When the illumination is turned off, the flows stop and the system relaxes to its original, equilibrium state.

At higher particle concentrations (32 % surface coverage), the experiment starts analogously to the low concentration setup: before irradiation with UV light, the particles are uniformly distributed over the fluid interface, albeit with a higher surface coverage (Fig. 4a). Switching on the UV irradiation in the area indicated in Fig. 4 also causes a collective flow toward the irradiated area. But, differently to the previous case, we do not observe any velocity reversal here. The flow drags the particles toward the center of the illuminated area, where they form a cluster (Fig. 4d). For the trajectories shown in Fig. 4d, corresponding to particles passing through the same location at different times after the UV was turned on, the velocity for each tracked trajectory is approximately constant (Fig. 4e). Comparing these velocities, it can be inferred that the magnitude increases with the time passed since the illumination was switched on (compare the slopes of the lines in Fig. 4e, from left to right). Alternatively, by considering a fixed time, e.g., $t = 4\,\mathrm {s}$, it can also be inferred that the velocity of a particle is larger when its distance from the illuminated area is larger. This inflow leads to a steady growth of the cluster; we do not observe particles escaping the cluster as long as the UV light is on. After turning off the UV light, the flows also stop; however, the dense cluster remains compact and merely breaks apart into larger pieces as the system relaxes. This fact can be interpreted as indicative of Marangoni flows that are sufficiently strong to push the particles so close as to foster van der Waals forces causing an essentially irreversible aggregation.

In summary, we can state that the observed collective flows depend significantly on the coverage by active particles. We now attempt to frame these findings into a simple theoretical model for activity-induced Marangoni flows.

### Theoretical model and analysis

Based on the experimental analysis, which pinpoints the activity-induced Marangoni flows as the most plausible source of the collective dynamics within the monolayer, the interpretation of the results is attempted within the theoretical framework proposed in Refs. [34, 38]. This is the simplest model for the collective motion of active particles by the self-induced Marangoni flow. Succinctly, it treats the monolayer as a continuum dragged by the ambient flow. This flow is described in the Stokes approximation, and it is driven by the chemical gradients determined according to the diffusion equation (Fig. 1f). The evolution within the monolayer is thus due to the hydrodynamic interactions between the particles (sourced by the interfacial response); even though the particles are lacking self-motility, the hydrodynamic interactions are long ranged ($\sim 1/r$, see, c.f., Eq. (3)) because of the induced Marangoni flows.

Thus, by adopting a coarse-grained approach to describe the large scale dynamics, we employ continuum fields that are assumed to vary slowly over the microscopic length scales (size of the particles, mean interparticle separation, etc.). The monolayer plane is identified with $z=0$; the vector $\mathbf {r}=(x,y)$ denotes the in-plane position and $\nabla := \left( \partial _x,\partial _y \right)$ the two-dimensional (2D) nabla operator in the monolayer plane. The areal number density of particles in the monolayer is given by the field $\varrho (\mathbf {r},t)$, which is the only relevant field because the particles are not intrinsically motile (i.e., there are no fields associated with a polar or a nematic order). We assume that there is no particle flux in or out of the monolayer; accordingly, $\varrho (\mathbf {r},t)$ is a conserved quantity and obeys the continuity equation

1 $$\begin{aligned} \frac{\partial \varrho }{\partial t} = - \nabla \cdot (\varrho \mathbf {u}) , \end{aligned}$$

as the particles are dragged by the ambient Marangoni flow $\mathbf {u}(\mathbf {r})$ induced by the activity of the particles, i.e., we assume that the only relevant cause of particle motion is this flow^4^. Notice that, although the Marangoni flow exists in the bulk of the fluid phases, the only relevant contribution to the dynamics is the 2D flow evaluated at the monolayer plane. A particularly important consequence is that, although the three-dimensional (3D) flow is incompressible, the projection of the flow onto the monolayer plane *is* compressible (see, c.f., Eq. (2)), and thus Eq. (1) does describe the emergence of an inhomogeneous distribution within the monolayer.

The activity of the particles is modeled as a source term in the concentration of a chemical involved in the reaction (e.g., $\hbox {O}_2$ as product, or $\hbox {H}_2\hbox {O}_2$ as reactant). One can assume that the molecular diffusion is much faster than the timescales associated with the collective motion, so that the distribution of chemical is adapted to the instantaneous configuration of the particles. Consequently, the concentration field can be found from the 3D Fick’s law with sources due to the active particles, i.e., by neglecting the time dependence as well as the drag by the ambient flows (low Péclet number), and solving a Poisson equation—see the color-coded field in Fig. 1f.

Since the surface tension of the interface depends on the local chemical composition, spatial variations of the surface tension develop due to the inhomogeneous distribution of chemicals in the fluid media. These gradients in surface tension translate to tangential forces at the interface (Marangoni stresses), which are transmitted to the fluids and generate the Marangoni flow. Assuming again that the flow adapts instantaneously to the particle configuration, the associated 3D velocity field is described by the Stokes equations for incompressible flow (low Reynolds number)—see the white streamlines in Fig. 1f.

Finally, one assumes that the surface tension changes linearly with the local concentration, which is a reasonable hypothesis if the range of variations is not too large. When this assumption is combined with the solutions of Fick’s law for the concentration of chemical and of Stokes’ equation for the flow, one ends up with a simple relationship between the particle density of the monolayer and the Marangoni flow at the monolayer plane (see Ref. [40] for the detailed derivation):

2 $$\begin{aligned} \mathbf {u} = - \nabla \varPhi , \;\; \nabla \cdot \mathbf {u} = \left\{ \begin{array}{ll} G \varrho (\mathbf {r}), &{}\quad \mathbf {r}\in \text {illuminated area,} \\ 0 , &{}\quad \mathbf {r}\not \in \text {illuminated area,} \end{array} \right. \nonumber \\ \end{aligned}$$

where the constant *G* (which can be either positive or negative) is proportional to the activity of the particles^5^.

Equations (1) and (2) form a closed system that allows one to obtain the monolayer density $\varrho (\mathbf {r},t)$. Notice that Eq. (2) means that the 2D flow at the monolayer is actually a Newtonian field; e.g., for $G<0$ the dynamics could be read as the (overdamped) collapse of a mass distribution under its own 2D gravity. The equations can be solved exactly, under the assumption of radial symmetry, for the initial condition of a homogeneous distribution, $\varrho (\mathbf {r},0)=\varrho _0$ (see “Appendix C”). In particular, for $G<0$ (flow “falling” into the illuminated area) the model predicts a 2D radial Marangoni flow in the non-illuminated area with radial component

3 $$\begin{aligned} u_r(r,t) = - \frac{A \mathrm {e}^{t/T}}{2\pi T r} , \end{aligned}$$

where $T=(|G|\varrho _0)^{-1}$ is a characteristic timescale set by the activity, and *A* is the area of the illuminated region.

One can compare this result with the quantitative observations presented in Fig. 4 as follows. During the observation time, the velocity of a particle does not change significantly (Fig. 4e), so that it would be given by its initial value: a particle located at a distance $R_i$ in the non-illuminated region at the time $t_i$ (measured from the onset of the illumination at $t = 0$) will have a velocity

4 $$\begin{aligned} V_i = - C_i \mathrm {e}^{t_i/T} , \qquad C_i = \frac{A}{2\pi T R_i}. \end{aligned}$$

This expression is used to fit the experimental data points, as shown in Fig. 4f; it can be seen that it provides a good approximation with the two fitting parameters $T\approx 11\,\mathrm {s}$, $C_i\approx 14.4\,\upmu \mathrm {m/s}$. With these numbers, Eq. (3) also predicts that the velocity of a particle is indeed approximately constant during the observation time (see Eq. (8) in “Appendix C”).

In spite of these results, there are discrepancies indicating that the experimental system is too rich to be fully captured by the simple theoretical model we have employed. First, the combination $T C_i \approx 158.4\,\upmu \mathrm {m}$ of the fitting parameters differs significantly from the prediction $A/2\pi R_i \approx 3.4\,\upmu \mathrm {m}$ for the value $R_i\approx 70\,\upmu \mathrm {m}$ of the trajectories depicted in Fig. 4e and the area $A\approx 1500\,\upmu \mathrm {m}$ of the illuminated region. Second, and more importantly, at given time *t* the velocity field according to Eq. (3) decays with the distance *r*, while the trajectories in Fig. 4e, which are probing this velocity field, actually show a flow that at fixed *t* increases in magnitude with *r*. This means, in particular, that this flow is compressible even in the non-illuminated regions, in disagreement with Eq. (2). Additionally, the theoretical model cannot explain the trajectories with velocity reversal depicted in Fig. 3d, because the sign of the velocity is fixed by the sign of the constant *G* (see Eq. (5) in “Appendix C”).

These arguments suggest that, contrary to the model assumptions, the activity that drives the flow is not located only in the illuminated region. The observation that, in the absence of surfactants, no collective motion is detected allows one to further conjecture that the surfactant is sensitive to the chemical reaction at the active particles, e.g., by desorbing from the $\hbox {TiO}_2$ particles when they are active or by reacting with produced intermediate reactive oxygen species. Accordingly, one may attempt to extend the theoretical model by allowing for additional sources of Marangoni flow, so that $\nabla \cdot \mathbf {u}\ne 0$ also in the non-illuminated region. In any case, these findings raise new questions, whose answer would require further experimental studies and complementary theoretical modeling.

## Conclusions

In conclusion, we have set up an experimental study to test the prediction of emergent collective dynamics driven by activity induced Marangoni flows, rather than self-propulsion, within a monolayer of chemically active particles at a fluid interface. The setup involves $\hbox {TiO}_2$ particles, which under UV illumination promote the photocatalytic decomposition of hydrogen peroxide in aqueous solutions. The particles are spherical by construction, so that they lack self-motility. We studied the configuration of particles sedimented at a quasi-flat silicon oil–water interface, in the presence of the nonionic surfactant TritonX in the aqueous phase. Upon UV illumination of a small central region, we have indeed observed the emergence of radial flows dragging the particles in the monolayer toward or away from the illuminated region, depending on the areal particle density within the monolayer.

Through a set of complementary experiments, we could identify activity-induced Marangoni flows as the most plausible cause of this dynamics and rule out various other a priori possible mechanisms (such as, e.g., phoretic interactions, thermal activity or UV response of the surfactant). Surprisingly, the dynamics exhibits a significant, qualitative change upon increasing the average areal density in the monolayer. At low densities, an initial transient inflow toward the illuminated region is replaced, within few seconds, by a monotonic outward flow, so that a growing area around the illuminated region emerges depleted of particles. In contrast, at large areal densities the inflow persists, leading to the steady growth of a particle cluster in the illuminated region.

The trajectories tracked for individual particles were analyzed within the framework of the simplest mean-field model for the dynamics driven by activity-induced Marangoni flow [40]. It turns out that the experimental setup is very rich and cannot be captured fully by the model. The most relevant conclusion is the finding that, contrary to expectations, the activity sources that drive the Marangoni flow are not localized in the illuminated area, but rather they also exist outside, although the photochemical activity in the illuminated area remains the ultimate driving factor. This led to the conjecture that the role played by the surfactant is more than just facilitating the entrapment of the particles at the interface or providing a more responsive interface. For instance, one can conceivably argue that the surfactant, which is not restricted to stay in the illuminated area, may be sensitive to the photochemical reaction at the $\hbox {TiO}_2$ particles. Future studies are required to understand and rationalize these findings.

## Figures and Tables

**Fig. 5:**
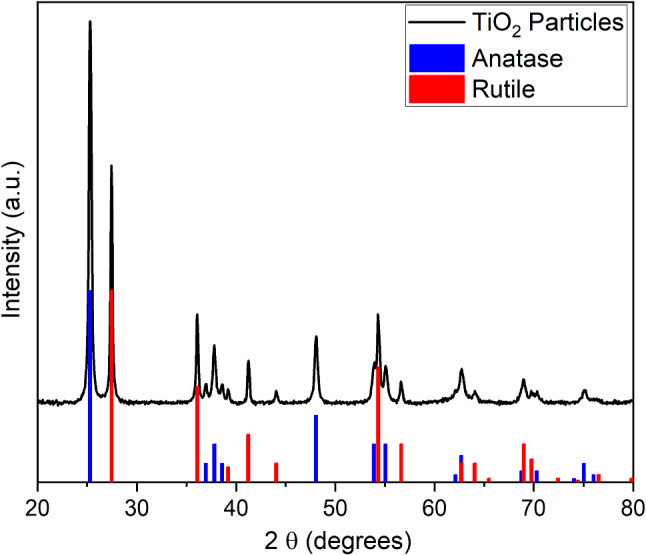
XRD of $\hbox {TiO}_2$ particles

**Fig. 8:**
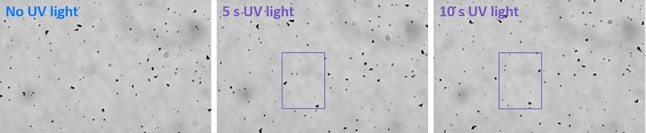
$\hbox {TiO}_2$ particles on oil–water interface in a solution of TritonX without $\hbox {H}_2\hbox {O}_2$. Left: before UV irradiation. Middle: after 5 s of UV irradiation. Right: after 10 s of UV irradiation

## Data Availability

This manuscript has associated data in a data repository. [Author’s comment: Data is available from the authors upon request.]

## Experimental details

### Materials

*Chemicals* All chemicals and solvents were used in analytical grade without any additional treatment: titanium (IV) isopropoxide (Alfa Aesar Co. Ltd.); dodecylamine (Fluka); silicone oil (Sigma-Aldrich).

*Particle synthesis* $\hbox {TiO}_{{2}}$ particles were synthesized via an improved version of a previously published method [43]. In brief, 0.18 mL of water was added to a mixture solution of 105 mL methanol and 45 mL acetonitrile. Then, 0.28 g of DDA was dissolved in the mixture and stirred for about 10 min. 1 mL of TTIP was added dropwisely and stirred for 12 hours, precipitates were removed and stirring was continued for 24 hours, when the process was repeated until no further precipitates were observed. Then, the particles were washed with ethanol three times and were calcined in a tubular furnace under nitrogen flow for 2 hours at 600 $^\circ$C.

*Surface functionalization* 24 $\times$ 24 mm glass slides were washed by sonication in acetone and ethanol and subsequently plasma cleaned. The surface functionalization was performed in the gas phase in a desiccator at 5 mbar for two days using 30 $\upmu$L hexadecyltrimethoxysilane per glass slide.

### Methods

*XRD* The phases of the $\hbox {TiO}_2$ particles were identified using XRD. Measurements were carried out with a Bruker 2D phaser in a 2$\varTheta$ range of 20$^\circ$–80$^\circ$. The XRD of $\hbox {TiO}_2$ particles (Fig. 5) only includes reflexes corresponding to the anatase and rutile phases, confirming the presence of a mixture of both polymorphic forms of $\hbox {TiO}_2$.

*Zeta potential measurements* The zeta potential was measured with a Malvern Zetasizer Nano ZSP in autotitration mode.

*Video recording* An inverted optical microscope (Carl Zeiss Microscope GmbH) equipped with a Zeiss Colibri LED lamp and a “N-Achroplan” 63x/0.95 M27 objective were used for observation. The wavelength of the UV light was 385 nm, the UV lamp power was fixed at 100% lamp intensity, corresponding to 315 mW. The particle behavior was recorded with a Zeiss camera (Axiocam 702 Mono) and a frame rate of 40 fps.

*Experiments on liquid interfaces* The cylindrical cell was filled with 800 $\upmu$L of a solution containing 1 % $\hbox {H}_2\hbox {O}_2$ and 0.05 wt% TritonX. A drop of silicon oil was placed on the hydrophobic bottom using a cannula. 10–50 $\upmu$L of particles dispersed in water (10 mg/mL) were added to the cell and given time to settle down before the observation with the microscope was started.

*Reference experiments on solid substrate* The experiments on solid substrate were carried out in the same cylindrical cell without the oil drop.

*Video analysis* Videos were analyzed using ImageJ 1.52p. Tracking was done with the Manual Tracking plug-in.

## Reference experiments

### Reference experiments on a fluid interface using passive $\hbox {SiO}_2$ particles

In the experiments with passive silica particles on the solid substrate and on the fluid interface, no motion induced by the UV light was observed (Fig. 6).

### Reference experiments on a fluid interface without surfactant addition

In these experiments we have observed that irradiation with UV light causes enhanced Brownian diffusion of the $\hbox {TiO}_2$ particles, but does not lead to collective dynamics. Some particles lift off and move upward out of focus (Fig. 7) if the UV intensity is sufficiently high.

### Reference experiments on a fluid interface without $\hbox {H}_2\hbox {O}_2$ addition

If $\hbox {H}_2\hbox {O}_2$ is not added to the solution, the UV light does not induce any particle motion (Fig. 8).

### Choosing a surfactant: zeta potential measurements

Zeta potential titrations were performed using Malvern Zetasizer Nano ZSP in autotitration mode using the attached Multi-Purpose Titrator. The titration was carried out adding the respective surfactant (SDS, CTAB and TritonX), and zeta potential values were recorded after every ml of the solution of surfactant was added.

### Reference experiments on solid substrates

Similarly to the observations in the case of $\hbox {TiO}_2$ particles on the oil–water interface without surfactant addition, when the interface is replaced by a wall the irradiation with UV light causes solely an enhanced Brownian diffusion and an upward motion of the particles, irrespective of the absence (Fig. 10) or presence (Fig. 11) of surfactant.

## Theoretical model and analysis

Here we solve Eqs. (1) and (2) under the assumption of radial symmetry in order to compare with the observations. We take the initial distribution of particles in the monolayer to be homogeneous, with density $\varrho _0$, and we approximate the illuminated area as a disk of fixed radius *L*. Therefore, the evolved density field will have radial symmetry about the center of the illuminated area. The velocity field of the Marangoni flow, which will only have a radial component $u_r(r,t)$, can be obtained easily by applying Gauss theorem to Eq. (2): thus, the flow outside of the illuminated area is given by

5 $$\begin{aligned} u_r(r,t) = \frac{G N(t)}{2\pi r}, \qquad r>L , \end{aligned}$$

where *N*(*t*) is the number of particles inside the illuminated area. By applying Gauss theorem over the illuminated area to Eq. (1) and combining with Eq. (5), one obtains

6 $$\begin{aligned} \frac{\partial N}{\partial t} = - 2\pi L u_r(L,t) \varrho (L,t) = - G N \varrho (L,t) . \end{aligned}$$

Assuming $G<0$, the velocity field (5) describes a “falling flow” into the illuminated region, so that all the particles approaching the rim of this region, and which determine the value $\varrho (L,t)$ of the density there, come from the non-illuminated region. But the 2D flow is incompressible in this region (see Eq. (2)), and consequently, the density at the rim does not change in time and it is equal to the homogeneous, initial one^6^, $\varrho (L,t)=\varrho _0$. Equation (6) then renders

7 $$\begin{aligned} N(t) = N_0 \mathrm {e}^{t/T}, \end{aligned}$$

where $T = 1/(|G|\,\varrho _0)$ is a characteristic timescale and $N_0 = \pi L^2 \varrho _0$ is the initial number of particles inside the illuminated area. Combining Eqs. (7) and (5), one arrives at the prediction in Eq. (3) with $A=\pi L^2$.

The distance *R*(*t*) of a particle to the center can be obtained by solving the equation of motion $dR/dt = u_r (R,t)$ with the initial condition $R(t_i)=R_i$. The initial velocity is given by Eq. (4), and the correction thereof can be estimated by Taylor expanding the velocity $V(t) = u_r(R(t),t)$ around the initial time:

8 $$\begin{aligned} \frac{V-V_i}{V_i} \approx \frac{1}{V_i} \left. \frac{\mathrm{d} V}{\mathrm{d}t}\right| _{t=t_i} (t-t_i) = \left( 1 - \frac{V_i T}{R_i} \right) \frac{t-t_i}{T} . \end{aligned}$$

With the value $T\approx 11\,\mathrm {s}$ obtained from the fit in Fig. 4f, this expression predicts that the relative error of approximating $V\approx V_i$ is at most $\approx 10\,\%$ for the trajectories depicted in Fig. 4e.

## Footnotes

1 A similar observation is predicted for the case of monolayers of isotropic active particle at a solid–liquid interface. However, in this case both the hydrodynamic interactions and the phoretic effective interaction (phoretic response of one particle to the chemical field produced by another particle) decay at least as $r^{-2}$ with the in-plane distance *r* between particles due to the boundary conditions imposed by the solid wall [36]. In contrast, the in-plane hydrodynamic interactions due to induced Marangoni flows have a significantly stronger effect due to their slow decay as $r^{-1}$ [34], which is formally analogous to gravity or electrostatics in two dimensions. It can have an attractive or repulsive character, depending on how the surface tension reacts to the activity of the particles.

2 When the system is also laterally confined, as in the case of a sessile drop containing active particles, the emergent collective dynamics driven by the self-induced Marangoni flows can be very rich; for example, Ref. [39] reported recently the observation of spontaneous symmetry breaking from a motionless monolayer to states of self-organized motion of particles and polar flow within the drop.

3 Albeit under the drawback of extremely weak (nm/s) Marangoni flows, thus requiring very long experiments, which are technically challenging.

4 The model can be easily extended to account for other driving forces, e.g., the thermal (Brownian) forces and the direct interactions between particles (e.g., steric repulsion), see Ref. [40].

5 More precisely, if the activity of a particle is quantified by the time rate *Q* at which the chemical reaction proceeds, then $G= Q b/[2\pi (\eta _1+\eta _2)(D_1+\lambda D_2)]$. Here *b* is the proportionality coefficient between the changes in the surface tension and the changes in the chemical concentration (in the linear approximation), $\eta _1, \eta _2$ are the viscosities of the fluid phases, $D_1, D_2$ are the diffusivities of the chemical in the fluids, and $\lambda$ is the ratio of solvabilities.
